# Facilitating a transition from compulsory detention of people who use drugs towards voluntary community-based drug dependence treatment and support services in Asia

**DOI:** 10.1186/s12954-015-0071-0

**Published:** 2015-10-16

**Authors:** Pascal Tanguay, Adeeba Kamarulzaman, Apinun Aramrattana, Alex Wodak, Nicholas Thomson, Robert Ali, Gino Vumbaca, Gloria Lai, Anand Chabungbam

**Affiliations:** Ozone Foundation, Bangkok, Thailand; University of Malaya, Kuala Lumpur, Malaysia; Department of Family Medicine, Chiang Mai University, Chiang Mai, Thailand; Alcohol and Drug Service, St Vincent’s Hospital, Sydney, Australia; University of Melbourne’s School of Population and Global Health, Melbourne, Australia; John Hopkins Bloomberg School of Public Health, Baltimore, United States; Australian National Advisory Council on Alcohol & Drugs (ANACAD), Adelaide, Australia; Australian National Council on Drugs (ANCD), Canberra, Australia; International Drug Policy Consortium, Bangkok, Thailand; Asian Network of People who Use Drugs, Bangkok, Thailand

**Keywords:** Drug dependence, Compulsory treatment, Community-based treatment, Planning, Drug policy reform, Health systems, Principles, Resource mobilization, People who use drugs

## Abstract

Evidence indicates that detention of people who use drugs in compulsory centers in the name of treatment is common in Cambodia, China, Indonesia, Lao PDR, Malaysia, Myanmar, Philippines, Thailand, and Vietnam. The expansion of such practices has been costly, has not generated positive health outcomes, and has not reduced supply or demand for illicit drugs. United Nations agencies have convened several consultations with government and civil society stakeholders in order to facilitate a transition to voluntary evidence- and community-based drug dependence treatment and support services.

In an effort to support such efforts, an informal group of experts proposes a three-step process to initiate and accelerate national-level transitions. Specifically, the working group recommends the establishment of a national multisectoral decision-making committee to oversee the development of national transition plans, drug policy reform to eliminate barriers to community-based drug dependence treatment and support services, and the integration of community-based drug dependence treatment in existing national health and social service systems.

In parallel, the working group recommends that national-level transitions should be guided by overarching principles, including ethics, human rights, meaningful involvement of affected communities, and client safety, as well as good governance, transparency, and accountability. The transition also represents an opportunity to review the roles and responsibilities of various agencies across the public health and public security sectors in order to balance the workload and ensure positive results.

The need to accelerate national-level transitions to voluntary community-based drug dependence treatment and support services is compelling—on economic, medical, sustainable community development, and ethical grounds—as extensively documented in the literature. In this context, the expert working group fully endorses initiation of a transition towards voluntary evidence- and community-based drug dependence treatment and support services across the region, as well as the steady scale-down of compulsory centers for drug users.

Components of voluntary community-based drug dependence treatment and support services are being implemented in Cambodia, China, Indonesia, Malaysia, and Thailand. However, significant technical and financial support will be required to be allocated from national budgets and by international development agencies in order to complete the transition and reduce the reliance on detention of people who use drugs in Asia.

## Background

The detention of people who use drugs (PWUD) remains a common response to drug use and drug dependence in Cambodia, China, Indonesia, Lao PDR, Malaysia, Myanmar, Philippines, Thailand, and Vietnam [1]. Although this is usually said to be implemented with the aim of treating and rehabilitating PWUD, the unspoken rationale justifying the scale-up of compulsory centers for drug users (CCDU) has included managing parental and community anxieties about drug use in settings where experience with drug use and evidence-based treatment is limited.

The exact number of people detained in the name of treatment in these countries is not known but unpublished data collected by the United Nations Office on Drugs and Crime (UNODC) indicates that almost half a million PWUD are detained in seven countries [2]. To date, the reliance on CCDU has resulted neither in sustained positive treatment outcomes nor in social rehabilitation [3] but rather has been associated with increased HIV risks [4, 5], added stigma and discrimination against PWUD, numerous serious documented human rights violations [6–15], and significant deviations from evidence-based best practices in drug dependence treatment [16–20]. In addition, there has been no significant or sustained decrease in drug production, trafficking, or use in Asia as a result of scaled-up compulsory detention and coercive approaches to drug treatment through CCDU [21]. Expanding the CCDU model has been costly and consumed considerable resources in an area where resources are extremely limited [22]. Evidence suggests that a great number of people currently forced into CCDU are not in need of clinical treatment for drug dependence, thereby exacerbating the cost burden [23, 24].

One significant obstacle to transitioning to community-based drug dependence and support services^1^ in many Asian countries is the increasing use and perceived problems associated with illicit drug use [25–29]. Significant advocacy efforts have generated increasing acceptance and implementation of opioid substitution therapy services across the region though coverage remains problematically low [30]. While psychosocial interventions are often helpful to address mild and moderate problems associated with amphetamine-type stimulants (ATS), the lack of a pharmacological substitute to effectively address the harms associated with ATS, including dependence, is a significant barrier.

Given the lack of evidence showing either health or criminogenic benefits of CCDU, some countries have initiated a transition to voluntary community-based drug dependence treatment. In order to stimulate dialogue and identify opportunities for national-level transitions towards voluntary community-based drug dependence treatment and support services, the United Nations Economic and Social Commission for Asia and the Pacific (UNESCAP), the UNODC Regional Office for Southeast Asia and the Pacific, and the Joint United Nations Program on HIV/AIDS (UNAIDS) Regional Support Team, Asia and the Pacific have convened a series of intergovernmental regional consultations^2^, with the support of the Australian National Council on Drugs, for a final high-level dialogue scheduled to be hosted in the Philippines in September 2015.

## Main text

In an effort to support this process, an informal group of experts^3^ has been established to prepare constructive advice to facilitate and support the operationalization of the national-level transitions and ultimately to develop a more effective and cost-efficient response to drug use and dependence across the region. Following a desk-based review of the evidence and a series of internal and external consultations with key partners and stakeholders, the expert working group proposes a three-step process to initiate national-level transitions.

As a first step, national multisectoral decision-making mechanism should be established with overall responsibility for the transition, including for the development, in consultation with key stakeholders from the public security, public health, and community sectors, including people who use drugs, of a comprehensive action plan or strategy that includes objectives, activities, outcomes, indicators, targets, budgets, timelines, and responsibilities. This tool can provide countries with a critical platform from which to coordinate the transition.

To promote voluntary access to drug treatment and support services, policy approaches to drug use and drug dependence need to shift away from criminalization and punishment to health- and rights-based drug policy measures. For example, instead of arrest, urine drug testing, and detention, governments should consider the adoption of programs that refer and divert people who use and are dependent on drugs to voluntary drug treatment and support services. Drug policy reforms to decriminalize drug use, as recommended by UNAIDS [31] and WHO [32], or depenalization to reduce the penalties associated with drug use should also be considered. Accordingly, national reviews to identify policies that restrict voluntary access to community-based drug dependence treatment and support services should be conducted as a critical step towards achieving an enabling policy environment for the transition.

Finally, reforms should be considered in order to develop and strengthen the various mechanisms underpinning implementation of drug dependence treatment and community-level support services, especially in the public health and public security sectors. These reforms should be accompanied by significant investments in the development of workforce capacity across multiple sectors, including among communities of PWUD. More specifically, across the region, opportunities for integrating voluntary community-based treatment and support services are available, especially where low-threshold health and social care services are already being delivered to PWUD. For example, in countries that have invested in harm reduction and scaling up comprehensive HIV prevention, treatment, care, and support services, a number of drug dependence treatment interventions can be integrated at existing service delivery outlets to maximize uptake and increase demand.

The three-step transition process described above should be informed and guided by a range of principles, including ethics, human rights, meaningful involvement of affected communities, and client safety, as well as good governance, transparency, and accountability. Compliance with these different good practice approaches will contribute to reducing the potential unintended negative consequences of the transition and maximize the chance of a successful transition.

The transition should also be considered an opportunity to more effectively balance the national workload associated with drug dependence between sectors, with particular attention for redefining the role of law enforcement in achieving both public security and public health objectives. After all, drug dependence is recognized as a chronic relapsing and remitting health condition that warrants medical attention, irrespective of other normative imperatives. In that context, overall national responses to individuals who use drugs and/or suffer from dependence should be integrated in national systems beyond criminal justice with greater authority delegated to medical professionals.

Finally, the transition towards voluntary community-based drug dependence treatment and services should result in better health outcomes at the client level. When clients are properly assessed and can voluntarily choose from a menu of drug dependence treatment and support service options, and where those options are available, accessible, and affordable to clients, better results will be generated. Recent evidence from Malaysia indicates a significant improvement in treatment outcomes when clients select drug dependence treatment modalities voluntarily [33].

Components of voluntary community-based drug dependence treatment and support services are currently being piloted, implemented, and evaluated in Cambodia, China, Indonesia, Malaysia, and Thailand [34]. For example, in China, law enforcement are diverting PWUD to the Ping An No. 1 Centre, established by AIDS Care China in 2014; in Cambodia, since 2011, the government, United Nations, and civil society organizations have worked together to provide 1200 PWID with voluntary access to community-based services aligned with international protocols in three provinces [35]; in Indonesia, PWUD have reported preferring accessing drug dependence treatment through Rumah Singgah PEKA’s community-based model rather than through government-operated CCDU; and in Malaysia, PWUD leaving from voluntary Cure and Care Centers have been shown to be considerably less likely to relapse compared to those leaving from CCDU [36]. Preliminary reports indicate significant positive results that will require closer scrutiny and evaluation in order to better inform the development of national plans.

## Conclusion

The need to formally initiate national-level transitions towards voluntary community-based drug dependence treatment and support services is compelling—on economic, medical, sustainable community development, and ethical grounds—as extensively documented in the literature. In this context, the expert working group fully endorses initiation of a transition towards voluntary evidence- and community-based drug dependence treatment and support services across the region, as well as the steady scale-down of CCDU.

The expert working group has formulated broad structural-level recommendations to support the formal operationalization of the transition in order to allow significant flexibilities for national level specificities to be addressed in a localized strategy. These structural recommendations recognize the need for national ownership and for responses tailored to the cultural context of each country.

However, implementation of the recommended structural actions and following the overarching principles presented here will likely be insufficient to effectively transition to community-based drug dependence treatment unless significant technical and financial support is allocated from national budgets and by international development agencies. In that respect, the recommendations formulated by the expert working group will be presented officially in Manila, the Philippines, in September 2015, in the context of the Third Regional Consultation on CCDU, in order to facilitate the development of transition plans fully owned at local and national levels and attract support from donor and technical support agencies for such efforts.

## Abbreviations

ATS: amphetamine-type stimulants
CCDU: compulsory center for drug users
PWUD: people who use drugs
UNAIDS: Joint United Nations Program on HIV/AIDS
UNESCAP: United Nations Economic and Social Commission for Asia and the Pacific
UNODC: United Nations Office on Drugs and Crime

## Footnotes

1 Support services include harm reduction as well as social support services such as employment and housing.

2 The First and Second Regional Consultations on Compulsory Centres for Drug Users (CCDU) were held in December 2010 in Bangkok, Thailand, and in October 2012 in Kuala Lumpur, Malaysia.

3 The expert working group is composed of: Prof. Adeeba Kamarulzaman, HIV and Infectious Disease Specialist at the University of Malayia, based out of Kuala Lumpur, Malaysia; Dr. Apinun Aramrattana, Head of the Department of Family Medicine at Chiang Mai University, in northern Thailand; Dr. Alex Wodak, Emeritus Consultant for the Alcohol and Drug Service at St. Vincent’s Hospital in Sydney, Australia; Dr. Nicholas Thomson, Senior Research Fellow at the University of Melbourne’s School of Population and Global Health and a current fellow of the International AIDS Society and the National Institute of Drug Abuse; Dr. Robert Ali, Executive Member of the Australian National Council on Drugs (ANCD), based in Adelaide, Australia; Mr. Gino Vumbaca, Executive Director of the Australian National Council on Drugs (ANCD), based in Canberra, Australia; Gloria Lai, Senior Policy Advisor at the International Drug Policy Consortium, based in Bangkok, Thailand; Mr. Anand Chabungbam is currently the Coordinator at the Asian Network of People who Use Drugs, based in Bangkok, Thailand. The working group operates with support from UNODC Regional Office for Southeast Asia and the Pacific and UNAIDS Regional Support Team, Asia and the Pacific, including secretarial support from consultant Mr. Pascal Tanguay.
